# Health-protective behaviour, social media usage and conspiracy belief during the COVID-19 public health emergency

**DOI:** 10.1017/S003329172000224X

**Published:** 2020-06-09

**Authors:** Daniel Allington, Bobby Duffy, Simon Wessely, Nayana Dhavan, James Rubin

**Affiliations:** King's College London, Digital Humanities, Strand, London, WC2R 2LS, UK

**Keywords:** Conspiracy beliefs, COVID-19, health-protective behaviours, public health, social media

## Abstract

**Background:**

Social media platforms have long been recognised as major disseminators of health misinformation. Many previous studies have found a negative association between health-protective behaviours and belief in the specific form of misinformation popularly known as ‘conspiracy theory’. Concerns have arisen regarding the spread of COVID-19 conspiracy theories on social media.

**Methods:**

Three questionnaire surveys of social media use, conspiracy beliefs and health-protective behaviours with regard to COVID-19 among UK residents were carried out online, one using a self-selecting sample (*N* = 949) and two using stratified random samples from a recruited panel (*N* = 2250, *N* = 2254).

**Results:**

All three studies found a negative relationship between COVID-19 conspiracy beliefs and COVID-19 health-protective behaviours, and a positive relationship between COVID-19 conspiracy beliefs and use of social media as a source of information about COVID-19. Studies 2 and 3 also found a negative relationship between COVID-19 health-protective behaviours and use of social media as a source of information, and Study 3 found a positive relationship between health-protective behaviours and use of broadcast media as a source of information.

**Conclusions:**

When used as an information source, unregulated social media may present a health risk that is partly but not wholly reducible to their role as disseminators of health-related conspiracy beliefs.

## Introduction

Conspiracism is the tendency to assume that major public events are secretly orchestrated by powerful and malevolent entities acting in concert (Douglas et al., 2019). The idea that such plotting explains social reality was influentially termed ‘the conspiracy theory of society’ by Popper (1969), and what Hofstadter termed ‘conspiratorial fantasies’ (1964) are now popularly referred to as ‘conspiracy theories’. Here, the more neutral ‘conspiracy beliefs’ is preferred. Online, such beliefs are now frequently offered as explanations of Coronavirus Disease 2019, or COVID-19. This outbreak of conspiracism is only the latest wave in an ongoing ‘deluge of conflicting information, misinformation and manipulated information on social media’ which some researchers have long argued ‘should be recognised as a global public-health threat’ (Larson, 2018, p. 309).

Multiple studies have found a link between medical conspiracy beliefs and reluctance to engage in health-protective behaviours with regard to vaccination or safer sex (Dunn et al., 2017; Goertzel, 2010; Grebe & Nattrass, 2011; Jolley & Douglas, 2014; Thorburn & Bogart, 2005; Zimmerman et al., 2005). This raises the possibility that the circulation of COVID-19 conspiracy beliefs might be associated with similar risks. Indeed, two recent studies have found a negative relationship between COVID-19 conspiracy beliefs and health-protective behaviours intended to help control the COVID-19 pandemic (Allington & Dhavan, 2020; Freeman et al., 2020).

YouTube and Facebook have been identified as major vectors for dissemination of conspiracy beliefs and misinformation, on medical and other topics (AVAAZ, 2020; Bora, Das, Barman, & Borah, 2018; Buchanan & Beckett, 2014; Byford, 2011, p. 11; Chaslot, 2017; Oi-Yee Li, Bailey, Huynh, & Chan, 2020; Pandey, Patni, Sing, Sood, & Singh, 2010; Pathak et al., 2015; Seymour, Getman, Saraf, Zhang, & Kalenderian, 2014; Sharma, Yadav, Yadav, & Ferdinand, 2017). Most studies of Twitter suggest that it plays a similar role (Broniatowski et al., 2018; Kouzy et al., 2020; Ortiz-Martínez & Jiménez-Arcia, 2017; Oyeyemi, Gabarron, & Wynn, 2014). But while social media misinformation is both pervasive and popular, its effects are hard to quantify, and it is unclear which groups are most susceptible to its influence (Wang, McKee, Torbica, & Stuckler, 2019).

In the UK, broadcast and print media are regulated (albeit by different mechanisms), while social media are unregulated. For example, when COVID-19 misinformation was propagated by David Icke and Brian Rose on the London Live television station, the owner of the station was sanctioned by the UK broadcasting regulator for disseminating content which ‘had the potential to cause significant harm to viewers’ (Ofcom, 2020, p. 16). However, similar content continues to circulate freely on social media platforms (Brennen, Simon, Howard, & Nielsen, 2020). While social media platforms can and do exercise editorial control over the content they disseminate, they appear to do so inconsistently (AVAAZ, 2020). Purveyors of conspiracy beliefs and other misinformation successfully exploit this situation for economic gain (CCDH, 2020; Scott, 2020).

We report on three online questionnaire surveys of engagement in COVID-19-specific health-protective behaviours, use of social media as a source of information about COVID-19, and COVID-19 conspiracy beliefs, defined as beliefs which entail that the COVID-19 public health critis was produced through intentional agency (whether through manufacture of the coronavirus or through deliberate exaggeration or incorrect attribution of negative health outcomes). The first and third surveys measured adherence to multiple conspiracy beliefs, while the second measured adherence to just one. The first and second measured media usage in very general terms, while the third separately measured informational reliance on legacy media as well as on specific social media platforms.

### Methods: data collection

Data for Study 1 were collected in partnership with CitizenMe. Invitations were sent to all members of a panel of UK residents aged 18 or more who had expressed an interest in answering surveys about COVID-19. Data for Studies 2 and 3 were collected in partnership with Ipsos-MORI, a member of the British Polling Council. The sampling frame was a recruited panel of UK adults aged 16–75. Stratified random samples were selected, with quotas employed to achieve national representativeness with regard to age within gender, region, working status, social grade and education, using census and mid-year estimates from the Office of National Statistics. Questionnaires were completed online. The data collection followed ethical and data protection procedures at King's College London and at the partner organisations. Fieldwork dates were 3–7 April 2020 for Study 1, 1–3 April 2020 for Study 2 and 20–22 May 2020 for Study 3.

Table 1 contains descriptive statistics for all samples. Where gender percentages do not sum to 100, this is because very small numbers of respondents did not self-identify as ‘Female’ or ‘Male’. Due to missing data, total *N* may vary throughout each study.

**Table 1.** Descriptive statistics, all three samples

Sample	*N*	Age (M)	Age (s.d.)	Female (%)	Male (%)
Study 1	949	36.35	10.49	68.28	31.51
Study 2	2250	45.47	17.66	51.33	48.22
Study 3	2254	43.93	16.11	49.87	49.73

### Methods: data analysis

Hypotheses regarding relationships between usage of or reliance on sources of information and either conspiracy beliefs or health-protective behaviours were tested using Mann–Whitney–Wilcoxon *U* tests, with effect sizes estimated using Vargha and Delaney's *A* with 95% confidence intervals (CIs) calculated through bootstrapping with 1000 replications, on the assumption that each sample can be treated as equivalent to a random sample. Hypotheses concerning conspiracy beliefs and health-protective behaviours were tested using Fisher's exact test both to calculate significance and to estimate odds ratios with a 95% CI (on the same assumption).

In all three studies, hypotheses were tested by treating conspiracy beliefs and health-protective behaviours both individually and in combination, with an aggregate variable to indicate whether a respondent held at least one conspiracy belief and another to indicate whether a respondent engaged in all health-protective behaviours. In Study 3, measurements of media use were both treated individually and aggregated by recoding ordinal variables as numeric variables and taking the mean, creating one aggregate variable for the legacy media and one aggregate variable for social media. Tests covering combinations of raw and aggregated variables are reported in tables in the online Supplementary materials, which are prefixed ‘S’.

Welch unequal variance *t* tests were used to test for effects of age and Fisher tests were used for effects of gender with regard to aggregate variables, again with a 95% CI. In Study 3, logistic regression models were used to control for the effects of multiple variables.

‘Don't know’ responses were treated as missing data. All tests were carried out using base R v. 3.6.1 (R Core Team, 2019), with the exception of Vargha and Delaney's *A*, which was calculated using the R library, rcompanion v. 2.3.21 (Mangiafico, 2020).

## Study 1

### Introduction

Respondents identified true statements from a list of six statements which included these three conspiracy beliefs: ‘The virus that causes COVID-19 was probably created in a laboratory’, ‘The symptoms of COVID-19 seem to be connected to 5G mobile network radiation’ and ‘The COVID-19 pandemic was planned by certain pharmaceutical corporations and government agencies’. Respondents answered the question ‘How do you find out what's going on in the world?’ using a five-point scale from ‘Always from major newspapers and/or TV channels (including online)’ to ‘Always from social media’, and were also asked to identify behaviours in which they were engaging from a list of six which included three health-protective behaviours (Table 2).

**Table 2.** Key variables, Study 1

Group	Questions
Conspiracy beliefs	CB.1.1 The virus that causes COVID-19 was probably created in a laboratory
	CB.1.2 The symptoms of COVID-19 seem to be connected to 5G mobile network radiation
	CB.1.3 The COVID-19 pandemic was planned by certain pharmaceutical corporations and government agencies
Health-protective behaviours	HPB.1.1 Spending as little time as possible outside your home
	HPB.1.2 Staying at least 2 m apart from anyone outside of your household
	HPB.1.3 Washing your hands more often, for 20 s
Information sources	IS.1.1 How do you find out what's going on in the world?

IS.1.1 Answer options:
1. Always from major newspapers and/or TV channels (including online) 2. More from major newspapers and/or TV channels (including online) than from social media 3. Equally from major newspapers and/or TV channels (including online) and from social media 4. More from social media than from major newspapers and/or TV channels (including online) 5. Always from social media 6. Don't know

### Hypotheses

H.1.1 A positive relationship between conspiracy belief and preference for social media over mainstream media

H.1.2 A negative relationship between health-protective behaviour and preference for social media over mainstream media

H.1.3 A negative relationship between conspiracy belief and health-protective behaviour

### Findings

The most commonly held conspiracy belief was CB.1.1 (a laboratory origin for the coronavirus). Those holding one or more conspiracy beliefs were very slightly younger, *t*(604.71) = −2.69, *p* = 0.007, 95% CI (−3.22 to −0.50), while those who engaged in health-protective behaviours were very slightly older, *t*(781.54) = 3.49, *p* < 0.001, 95% CI (1.06–3.78). Women were more likely to engage in all health-protective behaviours than men, *p* = 0.003, 95% CI (1.15–2.05), and there was no relationship between gender and conspiracy belief, *p* = 0.591, 95% CI (0.80–1.50). See online Supplementary Tables S.1.1–4.

There was a positive relationship between holding one or more conspiracy beliefs and preference for social media over legacy media as a general source of information, *U*(*N*1 = 266, *N*2 = 665) = 99 987.0, *p* = 0.001, 95% CI (0.52–0.60). Hypothesis H.1.1 is thus supported (online Supplementary Table S.1.5).

There was no relationship between engagement in all health-protective behaviours and preference for social media over legacy media as a general source of information, *U*(*N*1 = 580, *N*2 = 351) = 100 207.0, *p* = 0.680, 95% CI (0.46–0.53). There was also no relationship for individual health-protective behaviours. H.1.2 is thus unsupported (online Supplementary Table S.1.6).

There was a very strong negative relationship between holding one or more conspiracy beliefs and following all health-protective behaviours, *p* < 0.001, 95% CI (0.34–0.61). The strongest effects were observed for CB.1.2 (a connection between COVID-19 symptoms and 5G). H.1.3 is thus supported (online Supplementary Table S.1.7).

## Study 2

### Introduction

Respondents were asked whether the statement that ‘Coronavirus was probably made in a laboratory’ was true or false. They were also asked how frequently they were checking social media for information or updates about COVID-19 and whether they were engaging in each of several health-protective behaviours (Table 3).

**Table 3.** Key variables, Study 2

Group	Questions
Conspiracy belief	CB.2.1 Coronavirus was probably created in a laboratory
Health-protective behaviours	HPB.2.1 Hand washing more often, for 20 s
	HPB.2.2 Staying 2 m away from other people when outside your home
	HPB.2.3 Met up with friends or family outside your home^a^
	HPB.2.4 Gone to work or outside despite having symptoms that could be coronavirus^a^
	HPB.2.5 Had friends or family visit you at home^a^
Information sources	IS.2.1 How often, if at all, do you check social media (such as Facebook or Twitter) for information or updates about coronavirus?

a Reverse-coded.

IS.2.1 Answer options:
1. Once an hour or more 2. Several times a day 3. Daily 4. Less often 5. Never 6. I don't use social media 7. Don't know

Answer options 5 and 6 were treated as equivalent, producing an ordinal variable with five levels (as in Study 1).

### Hypotheses

H.2.1 A positive relationship between conspiracy belief and frequency of checking social media for information or updates about coronavirus

H.2.2 A negative relationship between health-protective behaviour and frequency of checking social media for information or updates about coronavirus

H.2.3 A negative relationship between conspiracy belief and health-protective behaviour

### Findings

Those holding the conspiracy belief were several years younger, *t*(950.90) = −6.44, *p* < 0.001, 95% CI (−7.54 to −4.02), while those who followed all health-protective behaviours were several years older, *t*(589.10) = 9.28, *p* < 0.001, 95% CI (6.99–10.74). Women were significantly more likely to engage in all health-protective behaviours than men, *p* < 0.001, 95% CI (1.65–2.62), and slightly less likely to hold the conspiracy belief, although this was not statistically significant, *p* = 0.214, 95% CI (0.70–1.09). See online Supplementary Tables S.2.1–4.

There was a significant positive relationship between holding the conspiracy belief and frequency of checking social media for news about COVID-19, *U*(*N*1 = 468, *N*2 = 1221) = 343 152, *p* < 0.001, 95% CI (0.57–0.63). H.2.1 is thus supported (online Supplementary Table S.2.5).

There was no relationship between engaging in all health-protective behaviours and frequency of checking social media for information or updates about COVID-19, *U*(*N*1 = 1785, *N*2 = 391) = 343 412.0, *p* = 0.611, 95% CI (0.46–0.52). However, there were significant negative relationships between frequency of checking social media for information or updates about COVID-19 and two individual health-protective behaviours, i.e. HPB.2.3 (avoiding social encounters outside the home) and HPB.2.4 (not going to work or outside with possible COVID-19 symptoms), as well as negative relationships for two further health-protective behaviours that fell just short of significance. H.2.2 thus receives qualified support (online Supplementary Table S.2.6).

There was a significant negative relationship between holding the conspiracy belief and engagement in all health-protective behaviours, *p* < 0.001, 95% CI (0.39–0.66). There was also a significant negative relationship between holding the conspiracy belief and engagement in each individual health-protective behaviour. H.2.3 is thus supported (online Supplementary Table S.2.7).

## Study 3

### Introduction

Respondents were asked about four of the same health-protective beliefs as in Study 2 and about a wider range of conspiracy beliefs than in either of the two previous studies. The media usage question was more detailed, inviting respondents to assess how much of their knowledge about COVID-19 was drawn from seven different sources, including four named social media platforms (Table 4).

**Table 4.** Key variables, study 3

Group	Questions
Conspiracy belief	CB.3.1 Coronavirus was probably created in a laboratory
	CB.3.2 The symptoms that most people blame on coronavirus appear to be linked to 5G network radiation
	CB.3.3 There is no hard evidence that coronavirus really exists
	CB.3.4 The number of people reported as dying from coronavirus is being deliberately exaggerated by the authorities
	CB.3.5 The current pandemic is part of a global effort to force everyone to be vaccinated whether they want to or not
Health-protective behaviours	HPB.3.1 Hand washing more often, for 20 s
	HPB.3.2 Staying 2 m away from other people when outside your home
	HPB.3.3 Gone to work or outside despite having symptoms that could be coronavirus^a^
	HPB3.4 Had friends or family visit you at home^a^
Information sources	Please tell us how much of what you know about coronavirus, if anything, comes from…
	IS.3.1 TV and radio broadcasters (including through their websites and online)
	IS.3.2 Newspapers and magazines (including through their websites and online)
	IS.3.3 YouTube
	IS.3.4 Facebook
	IS.3.5 WhatsApp
	IS.3.6 Twitter
	IS.3.7 Family or friends^b^

a Reverse-coded.

b Not aggregated.

IS.3.1-7 Answer options:
1. Nothing at all 2. Not very much 3. A fair amount 4. A great deal 5. Don't know

### Hypotheses

H.3.1 A negative relationship between conspiracy belief and reliance on legacy media (TV and radio broadcasters, newspapers and magazines) for knowledge about COVID-19

H.3.2 A positive relationship between conspiracy belief and reliance on the social media platforms (YouTube, Facebook, WhatsApp, Twitter) for knowledge about COVID-19

H.3.3 A positive relationship between health-protective behaviour and reliance on legacy media for knowledge about COVID-19

H.3.4 A negative relationship between health-protective behaviour and reliance on social media platforms for knowledge about COVID-19

H.3.5 A negative relationship between conspiracy belief and health-protective behaviour

No explicit hypothesis was formulated for information source IS.3.7 (‘Friends and family’), but the implicit hypothesis of an effect was tested for comparative purposes.

### Findings

Those holding one or more conspiracy beliefs were slightly younger, *t*(1597.01) = −4.33, *p* < 0.001, 95% CI (−5.02 to −1.89). Those who followed all health-protective behaviours were several years older, *t*(943.24) = 6.98, *p* < 0.001, 95% CI (3.99–7.12). Women in the sample were significantly more likely to follow all health-protective behaviours than men, *p* < 0.001, 95% CI (1.65–2.62), and slightly more likely to hold one or more conspiracy beliefs, although this was not statistically significant, *p* = 0.164, 95% CI (0.94–1.40). See online Supplementary Tables S.3.1–4.

There was a small but significant negative relationship between use of legacy media as a source of knowledge about COVID-19 and belief in one or more conspiracy theories, *U*(*N*1 = 884, *N*2 = 748) = 296 848.5, *p* < 0.001, 95% CI (0.42–0.48). However, the effect of television and radio considered alone was in most cases statistically significant, while the effect of newspapers and magazines considered alone was not. Hypothesis H.3.1 is thus supported, with the caveat that this is primarily due to the contribution made by broadcast media (online Supplementary Table S.3.5).

There was a strong positive relationship between use of social media platforms as sources of knowledge about COVID-19 and holding one or more conspiracy beliefs, *U*(*N*1 = 882, *N*2 = 748) = 424 640.0, *p* < 0.001, 95% CI (0.62–0.67). In almost all cases, there was also a significant positive relationship between each individual conspiracy belief and use of each platform. YouTube had the strongest association with conspiracy beliefs, followed by Facebook. The conspiracy beliefs most strongly associated with social media usage were CB.3.2 (a link between COVID-19 symptoms and 5G) and CB.3.3 (which cast doubt on the existence of the coronavirus). Hypothesis H.3.2 is thus supported (online Supplementary Table S.3.6).

There was also a smaller but significant positive relationship between holding one or more conspiracy beliefs and use of friends and family as a source of information about COVID-19, *U*(*N*1 = 878, *N*2 = 749) = 397 473.5, *p* < 0.001, 95% CI (0.57–0.63). The implicit hypothesis of an effect for reliance on this information source was thus supported (online Supplementary Table S.3.7).

There was a positive relationship between use of legacy media as a source of knowledge about COVID-19 and following all health-protective behaviours; however, this effect was small and of borderline significance *U*(*N*1 = 1610, *N*2 = 563) = 478 174.0, *p* < 0.046, 95% CI (0.50–0.56). The effect associated with TV and radio was more significant, *U*(*N*1 = 1601, *N*2 = 561) = 481 068.5, *p* = 0.006, 95% CI (0.51–0.56), while there was no individual effect for newspapers and magazines, *U*(*N*1 = 1594, *N*2 = 553) = 447 648.5, *p* = 0.564, 95% CI (0.48–0.54). Hypothesis H.3.3 is thus supported, with the caveat that this is largely due to the contribution made by broadcast media (online Supplementary Table S.3.8).

There was a much stronger and more significant negative relationship between use of social media as a source of knowledge about COVID-19 and engagement in health-protective behaviours, *U*(*N*1 = 1603, *N*2 = 563) = 342 191.5, *p* < 0.001, 95% CI (0.35–0.41). Except with regard to HPB.3.1 (hand-washing), where there was a weak negative effect that was only significant with regard to YouTube, WhatsApp and the aggregate social media variable, there was in every case a very highly significant negative relationship between use of each social media platform and each of the health-protective behaviours considered individually (*p* < 0.001). As with conspiracy beliefs, YouTube was the platform with the strongest association with this undesirable outcome. H.3.4 was thus supported (online Supplementary Table S.3.9).

There was a weaker but still significant negative relationship between use of friends and family as a source of knowledge about COVID-19 and engagement in all health-protective behaviours, *U*(*N*1 = 1601, *N*2 = 560) = 240 145.0, *p* < 0.001, 95% CI (0.40–0.47). The implicit hypothesis of an effect for this source of knowledge was thus supported (online Supplementary Table S.3.10).

There was a strong negative relationship between holding one or more conspiracy beliefs and engagement in all health-protective behaviours, *p* < 0.001, 95% CI (0.29–0.47). Except with regard to belief CB.3.1 (laboratory origin), this relationship was very highly significant with regard to every combination of behaviours and beliefs (*p* < 0.001). In relation to the aggregate variable, HPB.3.All, the strongest effect was associated with CB.3.3 (which cast doubt on the existence of the coronavirus), followed by CB.3.4 (which holds that deaths from the coronavirus have been exaggerated) and CB.3.2 (a link between COVID-19 symptoms and 5G). H.3.5 was thus supported, with the caveat that some conspiracy beliefs may have stronger effects on behaviour than others (online Supplementary Table S3.11).

Finally, a series of binomial logistic regression models were constructed to control for multiple variables as predictors of health-protective behaviour (Table 5). Conspiracy belief emerges as a more powerful predictor of health-protective behaviour non-engagement than gender, and as a more powerful predictor than age once controls for media usage are applied. Media usage – and especially social media usage – appears to be the most powerful predictor of all (at least as represented in these models), with age losing much of its predictive power once it is controlled for. (This contrasts with the finding of qualified or absent effects on behaviour in Studies 1 and 2. However, those studies did not collect such detailed data on media usage.)

**Table 5.** Binomial regression models, log-odds of following all health-protective behaviours (HPB.3.All)

	Est.	Low	High	s.e.	*t*	*p*
Demographics only (Res. df = 2173)						
(Intercept)	0.54	0.49	0.58	0.02	24.37	<0.001
Female	0.14	0.10	0.18	0.02	7.47	<0.001
Age	0.29	0.22	0.35	0.03	8.39	<0.001
Demographics and conspiracy belief (Res. df = 1591)						
(Intercept)	0.63	0.57	0.68	0.03	21.99	<0.001
Female	0.13	0.09	0.18	0.02	6.07	<0.001
Age	0.26	0.18	0.34	0.04	6.40	<0.001
CB.3.Any	−0.19	−0.23	−0.14	0.02	−8.51	<0.001
Demographics and media usage (Res. df = 2155)						
(Intercept)	0.60	0.53	0.67	0.03	17.20	<0.001
Female	0.12	0.09	0.16	0.02	6.63	<0.001
Age	0.13	0.06	0.21	0.04	3.58	<0.001
IS.3.LM	0.16	0.09	0.24	0.04	4.27	<0.001
IS.3.SM	−0.38	−0.47	−0.30	0.04	−8.89	<0.001
Demographics, conspiracy belief and media usage (Res. df = 1581)						
(Intercept)	0.67	0.58	0.75	0.04	15.50	<0.001
Female	0.11	0.07	0.16	0.02	5.24	<0.001
Age	0.10	0.02	0.19	0.04	2.30	0.021
CB.3.Any	−0.14	−0.18	−0.09	0.02	−6.09	<0.001
IS.3.LM	0.17	0.08	0.26	0.05	3.62	<0.001
IS.3.SM	−0.39	−0.49	−0.29	0.05	−7.44	<0.001

95% confidence intervals; predictor variables standardised to the range 0–1.

## Discussion

The studies reported here find a positive association between COVID-19 conspiracy beliefs and use of social media as a source of information about COVID-19, and a negative association between COVID-19 conspiracy beliefs and COVID-19-specific health-protective behaviours, with the strongest negative effects being associated with beliefs that imply that the coronavirus may not exist, that its lethality has been exaggerated, or that its symptoms may have a non-viral cause. In addition, Study 3 and, to a lesser extent, Study 2 find a negative association between use of social media as a source of information about COVID-19 and health-protective behaviours. Study 3 also finds a weaker negative association for use of friends and family as a source of information, and a positive association for use of legacy media, especially broadcast media. Findings are suggestive of a hierarchy of effects associated with different social media platforms, with YouTube appearing to be the most problematic, but all being associated with significant negative effects.

A consistent finding across the studies is that COVID-19 conspiracy beliefs are more likely to be held by younger respondents, and that health-protective behaviour was associated with both age and female gender. However, the regression analysis in Study 3 suggests that effects on health-protective behaviour associated with conspiracy belief and social media use are not accounted for by age or gender, and that some of the apparent effects of age may indeed be accounted for by differences in media usage. That is, it may be that older people are more likely to reject conspiracy beliefs and engage in health-protective behaviours in part because they make more use of broadcast media and less use of social media.

## Conclusions

All three studies suggest that conspiracy beliefs act to inhibit health-protective behaviours and that social media act as a vector for such beliefs. Studies 2 and 3 find further evidence of a link between social media and non-engagement in health-protective behaviours, and Study 3 finds evidence of an opposite relationship for legacy media, especially broadcast media. In the UK, broadcast media are subject to official regulation, and many print media platforms are subject to voluntary regulation, but social media are largely unregulated. One wonders how long this state of affairs can be allowed to persist while social media platforms continue to provide a worldwide distribution mechanism for medical misinformation.

## Limitations

Study 1 relies on a self-selecting sample, while Studies 2 and 3 rely on stratified random samples from a recruited panel. Moreover, all three studies rely on self-reports for measurement both of media usage and of compliance with health-protective behaviours.

## Supplementary material

For supplementary material accompanying this paper visit http://dx.doi.org/10.1017/S003329172000224X. click here to view supplementary material
