# Health-protective behaviour, social media usage and conspiracy belief during the COVID-19 public health emergency – CORRIGENDUM

**DOI:** 10.1017/S0033291721000593

**Published:** 2021-07

**Authors:** D. Allington, B. Duffy, S. Wessely, N. Dhavan, J. Rubin

**Affiliations:** 1 Department of Digital Humanities, King's College London, Strand, London, WC2R 2LS, UK; 2 Policy Institute, King's College London, Strand, London, WC2R 2LS, UK; 3 Department of Psychological Medicine, King's College London, Strand, London, WC2R 2LS, UK

This article was published in Psychological Medicine with some errors regarding table 5. The first version of this article incorrectly described the models in table 5 as logistic regression models, when in fact they are linear probability models.

The authors apologise for this error.
