# Supplementary material for: Health-protective behaviour, social media usage and conspiracy belief during the COVID-19 public health emergency
Source: Psychol Med. 2020 Jun 9:1–7. doi: 10.1017/S003329172000224X (PMC7298098; doi:10.1017/S003329172000224X)
Supplement: Supplementary file 1 [file S003329172000224Xsup001.docx]

Health-protective behaviour, social media usage, and conspiracy belief during the COVID-19 public health emergency

Supplementary material

03/06/2020

## Table S.1.1: Descriptive statistics by conspiracy beliefs and health-protective behaviours

| Variable | Value | N | % | Age (M) | Age (SD) | Female (%) | Male (%) |
| --- | --- | --- | --- | --- | --- | --- | --- |
| CB.1.1 | Yes | 230 | 24.24 | 35.62 | 9.26 | 71.74 | 27.83 |
|  | No | 719 | 75.76 | 36.58 | 10.84 | 67.18 | 32.68 |
| CB.1.2 | Yes | 49 | 5.16 | 34.90 | 8.73 | 75.51 | 24.49 |
|  | No | 900 | 94.84 | 36.43 | 10.57 | 67.89 | 31.89 |
| CB.1.3 | Yes | 93 | 9.80 | 34.61 | 8.60 | 74.19 | 25.81 |
|  | No | 856 | 90.20 | 36.54 | 10.66 | 67.64 | 32.13 |
| CB.1.Any | Yes | 273 | 28.77 | 35.02 | 9.05 | 69.60 | 30.04 |
|  | No | 676 | 71.23 | 36.88 | 10.97 | 67.75 | 32.10 |
| HPB.1.1 | Yes | 731 | 77.03 | 36.65 | 10.44 | 70.31 | 29.41 |
|  | No | 218 | 22.97 | 35.33 | 10.59 | 61.47 | 38.53 |
| HPB.1.2 | Yes | 736 | 77.56 | 36.81 | 10.59 | 69.97 | 29.76 |
|  | No | 213 | 22.44 | 34.76 | 9.99 | 62.44 | 37.56 |
| HPB.1.3 | Yes | 756 | 79.66 | 37.06 | 10.61 | 70.50 | 29.23 |
|  | No | 193 | 20.34 | 33.56 | 9.51 | 59.59 | 40.41 |
| HPB.1.All | Yes | 587 | 61.85 | 37.27 | 10.54 | 71.89 | 27.77 |
|  | No | 362 | 38.15 | 34.85 | 10.24 | 62.43 | 37.57 |

## Table S.1.2: Descriptive statistics by preference for social media over legacy media

| Variable | Value | N | % | Age (M) | Age (SD) | Female (%) | Male (%) |
| --- | --- | --- | --- | --- | --- | --- | --- |
| IS.1.1 | 1st (Lowest) | 307 | 32.35 | 38.63 | 11.17 | 68.40 | 31.60 |
| IS.1.1 | 2nd | 228 | 24.03 | 35.97 | 10.15 | 65.35 | 34.65 |
| IS.1.1 | 3rd | 226 | 23.81 | 35.07 | 10.54 | 70.35 | 29.65 |
| IS.1.1 | 4th | 143 | 15.07 | 34.69 | 8.98 | 72.03 | 26.57 |
| IS.1.1 | 5th (Highest) | 27 | 2.85 | 33.19 | 9.15 | 55.56 | 44.44 |

## Table S.1.3: Mean difference in age by aggregate conspiracy beliefs and health-protective behaviours

| Variable | DF | t | Est. | Low | High | p |
| --- | --- | --- | --- | --- | --- | --- |
| CB.1.Any | 604.71 | -2.69 | -1.86 | -0.50 | -3.22 | 0.007 |
| HPB.1.All | 781.54 | 3.49 | 2.42 | 3.78 | 1.06 | < 0.001 |

Welch unequal variances t-test, 95% confidence intervals, no effect = 0.00

## Table S.1.4: Odds ratios, female gender vs aggregate conspiracy beliefs and health-protective behaviours

| Variable | Est. | Low | High | p |
| --- | --- | --- | --- | --- |
| CB.1.Any | 1.09 | 0.80 | 1.50 | 0.591 |
| HPB.1.All | 1.54 | 1.15 | 2.05 | 0.003 |

Fisher’s exact test, 95% confidence intervals, no effect = 1.00

## Table S.1.5: Stochastic dominance of conspiracy beliefs with regard to preference for social media over legacy media

| Conspiracy belief | Source | Est. | Low | High | N1 | N2 | U | p |
| --- | --- | --- | --- | --- | --- | --- | --- | --- |
| CB.1.1 | IS.1.1 | 0.58 | 0.54 | 0.62 | 226 | 705 | 92319.5 | < 0.001 |
| CB.1.2 | IS.1.1 | 0.53 | 0.45 | 0.61 | 47 | 884 | 22027.5 | 0.470 |
| CB.1.3 | IS.1.1 | 0.54 | 0.47 | 0.60 | 92 | 839 | 41399.0 | 0.235 |
| CB.1.Any | IS.1.1 | 0.56 | 0.52 | 0.60 | 266 | 665 | 99987.0 | 0.001 |

Vargha and Delaney’s A, 95% confidence intervals, no effect = 0.50

Mann-Whitney-Wilcoxon U test

## Table S.1.6: Stochastic dominance of health-protective behaviours with regard to preference for social media over legacy media

| Health-protective behaviour | Source | Est. | Low | High | N1 | N2 | U | p |
| --- | --- | --- | --- | --- | --- | --- | --- | --- |
| HPB.1.1 | IS.1.1 | 0.50 | 0.46 | 0.55 | 721 | 210 | 76083.5 | 0.909 |
| HPB.1.2 | IS.1.1 | 0.52 | 0.48 | 0.56 | 727 | 204 | 76960.5 | 0.392 |
| HPB.1.3 | IS.1.1 | 0.48 | 0.43 | 0.52 | 747 | 184 | 65797.5 | 0.353 |
| HPB.1.All | IS.1.1 | 0.49 | 0.46 | 0.53 | 580 | 351 | 100207.0 | 0.680 |

Vargha and Delaney’s A, 95% confidence intervals, no effect = 0.50

Mann-Whitney-Wilcoxon U test

## Table S.1.7: Odds ratios, conspiracy beliefs vs health-protective behaviours

| Conspiracy belief | Health-protective behaviour | Est. | Low | High | p |
| --- | --- | --- | --- | --- | --- |
| CB.1.1 | HPB.1.1 | 0.82 | 0.58 | 1.18 | 0.280 |
| CB.1.1 | HPB.1.2 | 0.70 | 0.49 | 1.00 | 0.046 |
| CB.1.1 | HPB.1.3 | 0.78 | 0.54 | 1.14 | 0.188 |
| CB.1.1 | HPB.1.All | 0.66 | 0.48 | 0.91 | 0.008 |
| CB.1.2 | HPB.1.1 | 0.49 | 0.26 | 0.96 | 0.023 |
| CB.1.2 | HPB.1.2 | 0.43 | 0.23 | 0.84 | 0.008 |
| CB.1.2 | HPB.1.3 | 0.42 | 0.22 | 0.81 | 0.006 |
| CB.1.2 | HPB.1.All | 0.37 | 0.19 | 0.69 | < 0.001 |
| CB.1.3 | HPB.1.1 | 0.79 | 0.48 | 1.35 | 0.364 |
| CB.1.3 | HPB.1.2 | 0.34 | 0.21 | 0.54 | < 0.001 |
| CB.1.3 | HPB.1.3 | 0.49 | 0.30 | 0.82 | 0.004 |
| CB.1.3 | HPB.1.All | 0.47 | 0.30 | 0.74 | < 0.001 |
| CB.1.Any | HPB.1.1 | 0.60 | 0.43 | 0.84 | 0.002 |
| CB.1.Any | HPB.1.2 | 0.42 | 0.30 | 0.58 | < 0.001 |
| CB.1.Any | HPB.1.3 | 0.47 | 0.33 | 0.66 | < 0.001 |
| CB.1.Any | HPB.1.All | 0.46 | 0.34 | 0.61 | < 0.001 |

Fisher’s exact test, 95% confidence intervals, no effect = 1.00

## Table S.2.1: Descriptive statistics by conspiracy belief and aggregate health-protective behaviours

| Variable | Value | N | % | Age (M) | Age (SD) | Female (%) | Male (%) |
| --- | --- | --- | --- | --- | --- | --- | --- |
| CB.2.1 | Yes | 474 | 24.60 | 41.62 | 16.08 | 47.78 | 52.01 |
|  | No | 1236 | 49.36 | 47.40 | 17.95 | 51.17 | 48.42 |
| HPB.2.1 | Yes | 2085 | 92.61 | 46.05 | 17.63 | 52.42 | 47.19 |
|  | No | 149 | 6.76 | 38.64 | 16.45 | 36.05 | 62.59 |
| HPB.2.2 | Yes | 2133 | 94.25 | 46.14 | 17.55 | 52.27 | 47.26 |
|  | No | 98 | 5.05 | 33.51 | 15.30 | 32.29 | 67.71 |
| HPB.2.3 | Yes | 2081 | 92.78 | 46.31 | 17.51 | 52.50 | 47.07 |
|  | No | 151 | 6.59 | 35.87 | 16.65 | 36.00 | 63.33 |
| HPB.2.4 | Yes | 2132 | 94.46 | 46.27 | 17.53 | 52.37 | 47.16 |
|  | No | 88 | 4.05 | 30.57 | 13.18 | 26.44 | 73.56 |
| HPB.2.5 | Yes | 2107 | 93.42 | 46.06 | 17.49 | 52.28 | 47.25 |
|  | No | 115 | 5.31 | 38.82 | 19.00 | 39.47 | 60.53 |
| HPB.2.All | Yes | 1817 | 80.25 | 47.21 | 17.33 | 54.65 | 44.91 |
|  | No | 400 | 18.35 | 38.35 | 17.28 | 36.68 | 62.81 |

## Table S.2.2: Descriptive statistics by frequency of checking social media for news about COVID-19

| Variable | Value | N | % | Age (M) | Age (SD) | Female (%) | Male (%) |
| --- | --- | --- | --- | --- | --- | --- | --- |
| IS.2.1 | 1st (Lowest) | 598 | 26.13 | 55.47 | 15.92 | 43.65 | 56.19 |
| IS.2.1 | 2nd | 337 | 15.09 | 44.79 | 17.92 | 47.18 | 52.23 |
| IS.2.1 | 3rd | 541 | 24.38 | 42.38 | 17.05 | 53.70 | 45.37 |
| IS.2.1 | 4th | 592 | 25.48 | 41.61 | 16.23 | 56.93 | 42.74 |
| IS.2.1 | 5th (Highest) | 136 | 6.56 | 35.28 | 14.19 | 58.82 | 41.18 |

## Table S.2.3: Mean difference in age by conspiracy belief and aggregate health-protective behaviours

| Variable | DF | t | Est. | Low | High | p |
| --- | --- | --- | --- | --- | --- | --- |
| CB.2.1 | 950.09 | -6.44 | -5.78 | -4.02 | -7.54 | < 0.001 |
| HPB.2.All | 589.10 | 9.28 | 8.86 | 10.74 | 6.99 | < 0.001 |

Welch unequal variances t-test, 95% confidence intervals, no effect = 0.00

## Table S.2.4: Odds ratios, female gender vs conspiracy belief and aggregate health-protective behaviours

| Variable | Est. | Low | High | p |
| --- | --- | --- | --- | --- |
| CB.2.1 | 0.87 | 0.70 | 1.09 | 0.214 |
| HPB.2.All | 2.08 | 1.65 | 2.62 | < 0.001 |

Fisher’s exact test, 95% confidence intervals, no effect = 1.00

## Table S.2.5: Stochastic dominance of conspiracy belief with regard to frequency of checking social media for news about COVID-19

| Conspiracy belief | Source | Est. | Low | High | N1 | N2 | U | p |
| --- | --- | --- | --- | --- | --- | --- | --- | --- |
| CB.2.1 | IS.2.1 | 0.6 | 0.57 | 0.63 | 468 | 1221 | 343152 | < 0.001 |

Vargha and Delaney’s A, 95% confidence intervals, no effect = 0.50

Mann-Whitney-Wilcoxon U test

## Table S.2.6: Stochastic dominance of health-protective behaviours with regard to frequency of checking social media for news about COVID-19

| Health-protective behaviour | Source | Est. | Low | High | N1 | N2 | U | p |
| --- | --- | --- | --- | --- | --- | --- | --- | --- |
| HPB.2.1 | IS.2.1 | 0.55 | 0.50 | 0.59 | 2043 | 147 | 163542.5 | 0.063 |
| HPB.2.2 | IS.2.1 | 0.49 | 0.44 | 0.55 | 2093 | 96 | 98417.5 | 0.728 |
| HPB.2.3 | IS.2.1 | 0.43 | 0.38 | 0.47 | 2045 | 145 | 126702.0 | 0.003 |
| HPB.2.4 | IS.2.1 | 0.44 | 0.37 | 0.49 | 2092 | 86 | 78185.0 | 0.034 |
| HPB.2.5 | IS.2.1 | 0.45 | 0.40 | 0.50 | 2068 | 112 | 103782.5 | 0.056 |
| HPB.2.All | IS.2.1 | 0.49 | 0.46 | 0.52 | 1785 | 391 | 343412.0 | 0.611 |

Vargha and Delaney’s A, 95% confidence intervals, no effect = 0.50

Mann-Whitney-Wilcoxon U test

## Table S.2.7: Odds ratios, conspiracy belief vs health-protective behaviours

| Conspiracy belief | Health-protective behaviour | Est. | Low | High | p |
| --- | --- | --- | --- | --- | --- |
| CB.2.1 | HPB.2.1 | 0.55 | 0.37 | 0.81 | 0.002 |
| CB.2.1 | HPB.2.2 | 0.52 | 0.32 | 0.85 | 0.008 |
| CB.2.1 | HPB.2.3 | 0.51 | 0.34 | 0.76 | < 0.001 |
| CB.2.1 | HPB.2.4 | 0.33 | 0.20 | 0.55 | < 0.001 |
| CB.2.1 | HPB.2.5 | 0.44 | 0.28 | 0.70 | < 0.001 |
| CB.2.1 | HPB.2.All | 0.50 | 0.39 | 0.66 | < 0.001 |

Fisher’s exact test, 95% confidence intervals, no effect = 1.00

## Table S.3.1: Descriptive statistics by conspiracy beliefs and health-protective behaviours

| Variable | Value | N | % | Age (M) | Age (SD) | Female (%) | Male (%) |
| --- | --- | --- | --- | --- | --- | --- | --- |
| CB.3.1 | Yes | 642 | 30.14 | 43.83 | 15.99 | 50.70 | 48.99 |
|  | No | 974 | 40.60 | 42.83 | 16.24 | 47.28 | 52.31 |
| CB.3.2 | Yes | 174 | 8.13 | 31.47 | 12.68 | 47.13 | 52.87 |
|  | No | 1820 | 78.75 | 45.52 | 15.85 | 49.23 | 50.28 |
| CB.3.3 | Yes | 155 | 7.32 | 31.92 | 12.70 | 44.16 | 55.19 |
|  | No | 1887 | 81.92 | 45.27 | 15.95 | 49.63 | 49.95 |
| CB.3.4 | Yes | 287 | 13.74 | 37.78 | 14.80 | 49.30 | 50.70 |
|  | No | 1594 | 68.87 | 45.31 | 16.03 | 48.81 | 50.75 |
| CB.3.5 | Yes | 271 | 12.67 | 35.70 | 14.48 | 48.70 | 50.93 |
|  | No | 1627 | 69.82 | 45.76 | 15.92 | 49.05 | 50.52 |
| CB.3.Any | Yes | 887 | 41.47 | 41.45 | 16.23 | 49.89 | 49.89 |
|  | No | 749 | 30.42 | 44.91 | 15.96 | 46.39 | 53.07 |
| HPB.3.1 | Yes | 2021 | 89.84 | 44.34 | 16.15 | 51.24 | 48.36 |
|  | No | 192 | 8.29 | 41.03 | 15.21 | 37.17 | 62.30 |
| HPB.3.2 | Yes | 2095 | 92.31 | 44.56 | 15.96 | 51.17 | 48.40 |
|  | No | 106 | 5.03 | 35.34 | 15.51 | 30.19 | 69.81 |
| HPB.3.3 | Yes | 2081 | 91.86 | 44.86 | 15.98 | 51.01 | 48.56 |
|  | No | 131 | 6.16 | 32.21 | 12.60 | 37.40 | 62.60 |
| HPB.3.4 | Yes | 1882 | 83.19 | 45.01 | 15.71 | 52.18 | 47.39 |
|  | No | 323 | 14.36 | 39.49 | 17.18 | 39.94 | 59.75 |
| HPB.3.All | Yes | 1615 | 71.29 | 45.49 | 15.70 | 53.81 | 45.69 |
|  | No | 564 | 25.08 | 39.93 | 16.49 | 39.08 | 60.75 |

## Table S.3.2: Descriptive statistics by reported level of knowledge about COVID-19 from each source

| Variable | Value | N | % | Age (M) | Age (SD) | Female (%) | Male (%) |
| --- | --- | --- | --- | --- | --- | --- | --- |
| IS.3.1 | 1st (Lowest) | 120 | 5.36 | 41.89 | 16.36 | 52.50 | 47.50 |
| IS.3.1 | 2nd | 263 | 11.13 | 39.71 | 15.48 | 48.67 | 50.57 |
| IS.3.1 | 3rd | 1010 | 45.32 | 43.84 | 16.16 | 52.28 | 47.22 |
| IS.3.1 | 4th (Highest) | 839 | 37.10 | 45.79 | 15.92 | 47.19 | 52.57 |
| IS.3.2 | 1st (Lowest) | 449 | 20.82 | 47.22 | 14.90 | 46.99 | 52.56 |
| IS.3.2 | 2nd | 485 | 21.41 | 44.72 | 15.19 | 48.55 | 50.62 |
| IS.3.2 | 3rd | 900 | 40.43 | 43.24 | 16.73 | 53.01 | 46.77 |
| IS.3.2 | 4th (Highest) | 382 | 15.53 | 41.17 | 16.28 | 47.24 | 52.49 |
| IS.3.3 | 1st (Lowest) | 1319 | 58.29 | 48.47 | 14.87 | 52.47 | 47.08 |
| IS.3.3 | 2nd | 437 | 19.11 | 40.58 | 15.90 | 48.17 | 51.61 |
| IS.3.3 | 3rd | 294 | 12.89 | 34.46 | 14.20 | 44.56 | 55.10 |
| IS.3.3 | 4th (Highest) | 139 | 6.54 | 29.84 | 11.47 | 37.68 | 61.59 |
| IS.3.4 | 1st (Lowest) | 1014 | 44.83 | 48.92 | 15.54 | 44.52 | 55.08 |
| IS.3.4 | 2nd | 685 | 29.82 | 42.26 | 15.68 | 54.47 | 45.10 |
| IS.3.4 | 3rd | 388 | 17.47 | 37.46 | 14.26 | 56.19 | 43.30 |
| IS.3.4 | 4th (Highest) | 130 | 6.12 | 32.68 | 11.96 | 46.51 | 53.49 |
| IS.3.5 | 1st (Lowest) | 1457 | 64.55 | 47.67 | 15.57 | 49.21 | 50.52 |
| IS.3.5 | 2nd | 431 | 19.00 | 39.40 | 14.87 | 54.88 | 44.65 |
| IS.3.5 | 3rd | 212 | 9.36 | 32.63 | 12.67 | 45.50 | 53.55 |
| IS.3.5 | 4th (Highest) | 87 | 4.04 | 30.32 | 11.02 | 40.23 | 58.62 |
| IS.3.6 | 1st (Lowest) | 1350 | 60.49 | 48.32 | 15.29 | 50.00 | 49.70 |
| IS.3.6 | 2nd | 418 | 17.45 | 39.35 | 15.22 | 51.32 | 47.96 |
| IS.3.6 | 3rd | 278 | 12.36 | 34.83 | 13.70 | 48.74 | 50.54 |
| IS.3.6 | 4th (Highest) | 116 | 5.27 | 30.27 | 11.38 | 34.48 | 65.52 |
| IS.3.7 | 1st (Lowest) | 316 | 13.33 | 49.62 | 14.85 | 37.03 | 62.03 |
| IS.3.7 | 2nd | 908 | 40.36 | 46.79 | 15.43 | 51.05 | 48.51 |
| IS.3.7 | 3rd | 810 | 36.17 | 40.99 | 16.09 | 53.84 | 46.16 |
| IS.3.7 | 4th (Highest) | 196 | 8.93 | 34.43 | 14.25 | 48.47 | 50.51 |

## Table S.3.3: Mean difference in age by aggregate conspiracy beliefs and health-protective behaviours

| Variable | DF | t | Est. | Low | High | p |
| --- | --- | --- | --- | --- | --- | --- |
| CB.3.Any | 1597.01 | -4.33 | -3.45 | -1.89 | -5.02 | < 0.001 |
| HPB.3.All | 943.24 | 6.98 | 5.56 | 7.12 | 3.99 | < 0.001 |

Welch unequal variances t-test, 95% confidence intervals, no effect = 0.00

## Table S.3.4: Odds ratios, female gender vs conspiracy beliefs and health-protective behaviours

| Variable | Est. | Low | High | p |
| --- | --- | --- | --- | --- |
| CB.3.Any | 1.15 | 0.94 | 1.40 | 0.164 |
| HPB.3.All | 1.82 | 1.49 | 2.22 | < 0.001 |

Fisher’s exact test, 95% confidence intervals, no effect = 1.00

## Table S.3.5: Stochastic dominance of conspiracy beliefs with regard to reported level of knowledge about COVID-19 from each legacy media source

| Conspiracy belief | Source | Est. | Low | High | N1 | N2 | U | p |
| --- | --- | --- | --- | --- | --- | --- | --- | --- |
| CB.3.1 | IS.3.1 | 0.48 | 0.45 | 0.51 | 637 | 971 | 297664.0 | 0.168 |
| CB.3.1 | IS.3.2 | 0.47 | 0.44 | 0.50 | 630 | 963 | 285355.5 | 0.036 |
| CB.3.1 | IS.3.LM | 0.47 | 0.44 | 0.50 | 640 | 973 | 293353.0 | 0.044 |
| CB.3.2 | IS.3.1 | 0.38 | 0.34 | 0.42 | 172 | 1813 | 119522.0 | < 0.001 |
| CB.3.2 | IS.3.2 | 0.51 | 0.47 | 0.56 | 172 | 1798 | 158357.0 | 0.583 |
| CB.3.2 | IS.3.LM | 0.44 | 0.40 | 0.49 | 174 | 1816 | 139995.5 | 0.011 |
| CB.3.3 | IS.3.1 | 0.41 | 0.36 | 0.46 | 155 | 1875 | 120015.5 | < 0.001 |
| CB.3.3 | IS.3.2 | 0.53 | 0.49 | 0.58 | 153 | 1861 | 151688.5 | 0.157 |
| CB.3.3 | IS.3.LM | 0.47 | 0.42 | 0.52 | 155 | 1883 | 137405.0 | 0.214 |
| CB.3.4 | IS.3.1 | 0.40 | 0.36 | 0.43 | 284 | 1588 | 178641.0 | < 0.001 |
| CB.3.4 | IS.3.2 | 0.48 | 0.44 | 0.52 | 283 | 1576 | 213014.5 | 0.207 |
| CB.3.4 | IS.3.LM | 0.43 | 0.39 | 0.47 | 286 | 1591 | 193856.5 | < 0.001 |
| CB.3.5 | IS.3.1 | 0.43 | 0.40 | 0.46 | 269 | 1618 | 188200.0 | < 0.001 |
| CB.3.5 | IS.3.2 | 0.50 | 0.46 | 0.54 | 266 | 1606 | 212866.5 | 0.925 |
| CB.3.5 | IS.3.LM | 0.47 | 0.43 | 0.51 | 271 | 1623 | 206596.0 | 0.101 |
| CB.3.Any | IS.3.1 | 0.44 | 0.41 | 0.46 | 881 | 746 | 287775.0 | < 0.001 |
| CB.3.Any | IS.3.2 | 0.47 | 0.45 | 0.50 | 868 | 747 | 307658.0 | 0.063 |
| CB.3.Any | IS.3.LM | 0.45 | 0.42 | 0.48 | 884 | 748 | 296848.5 | < 0.001 |

Vargha and Delaney’s A, 95% confidence intervals, no effect = 0.50

Mann-Whitney-Wilcoxon U test

## Table S.3.6: Stochastic dominance of conspiracy beliefs with regard to reported level of knowledge about COVID-19 from each social media source

| Conspiracy belief | Source | Est. | Low | High | N1 | N2 | U | p |
| --- | --- | --- | --- | --- | --- | --- | --- | --- |
| CB.3.1 | IS.3.3 | 0.57 | 0.55 | 0.60 | 624 | 952 | 339616.5 | < 0.001 |
| CB.3.1 | IS.3.4 | 0.58 | 0.55 | 0.61 | 627 | 968 | 351191.5 | < 0.001 |
| CB.3.1 | IS.3.5 | 0.55 | 0.53 | 0.58 | 622 | 955 | 328170.5 | < 0.001 |
| CB.3.1 | IS.3.6 | 0.50 | 0.48 | 0.53 | 615 | 946 | 292173.0 | 0.867 |
| CB.3.1 | IS.3.SM | 0.57 | 0.54 | 0.60 | 637 | 971 | 351915.5 | < 0.001 |
| CB.3.2 | IS.3.3 | 0.78 | 0.75 | 0.82 | 171 | 1778 | 238632.0 | < 0.001 |
| CB.3.2 | IS.3.4 | 0.73 | 0.69 | 0.77 | 172 | 1794 | 225575.5 | < 0.001 |
| CB.3.2 | IS.3.5 | 0.71 | 0.67 | 0.76 | 167 | 1772 | 211339.0 | < 0.001 |
| CB.3.2 | IS.3.6 | 0.69 | 0.64 | 0.73 | 165 | 1760 | 199886.0 | < 0.001 |
| CB.3.2 | IS.3.SM | 0.80 | 0.76 | 0.83 | 174 | 1809 | 250419.0 | < 0.001 |
| CB.3.3 | IS.3.3 | 0.76 | 0.72 | 0.80 | 154 | 1842 | 216909.0 | < 0.001 |
| CB.3.3 | IS.3.4 | 0.71 | 0.67 | 0.76 | 152 | 1862 | 202071.0 | < 0.001 |
| CB.3.3 | IS.3.5 | 0.70 | 0.65 | 0.74 | 147 | 1842 | 188807.5 | < 0.001 |
| CB.3.3 | IS.3.6 | 0.69 | 0.65 | 0.74 | 149 | 1821 | 187934.0 | < 0.001 |
| CB.3.3 | IS.3.SM | 0.78 | 0.74 | 0.82 | 155 | 1877 | 227154.0 | < 0.001 |
| CB.3.4 | IS.3.3 | 0.67 | 0.64 | 0.71 | 280 | 1560 | 293997.5 | < 0.001 |
| CB.3.4 | IS.3.4 | 0.65 | 0.61 | 0.68 | 284 | 1575 | 288996.0 | < 0.001 |
| CB.3.4 | IS.3.5 | 0.60 | 0.57 | 0.64 | 279 | 1563 | 261993.0 | < 0.001 |
| CB.3.4 | IS.3.6 | 0.61 | 0.57 | 0.64 | 273 | 1551 | 257730.5 | < 0.001 |
| CB.3.4 | IS.3.SM | 0.68 | 0.65 | 0.72 | 286 | 1587 | 310710.5 | < 0.001 |
| CB.3.5 | IS.3.3 | 0.73 | 0.70 | 0.76 | 265 | 1589 | 308240.0 | < 0.001 |
| CB.3.5 | IS.3.4 | 0.67 | 0.64 | 0.71 | 268 | 1608 | 289251.5 | < 0.001 |
| CB.3.5 | IS.3.5 | 0.67 | 0.64 | 0.70 | 263 | 1589 | 279273.5 | < 0.001 |
| CB.3.5 | IS.3.6 | 0.64 | 0.60 | 0.68 | 261 | 1578 | 264327.0 | < 0.001 |
| CB.3.5 | IS.3.SM | 0.74 | 0.71 | 0.78 | 271 | 1617 | 326231.0 | < 0.001 |
| CB.3.Any | IS.3.3 | 0.65 | 0.62 | 0.67 | 862 | 738 | 410893.0 | < 0.001 |
| CB.3.Any | IS.3.4 | 0.62 | 0.60 | 0.65 | 869 | 745 | 404703.5 | < 0.001 |
| CB.3.Any | IS.3.5 | 0.60 | 0.58 | 0.62 | 858 | 742 | 383238.0 | < 0.001 |
| CB.3.Any | IS.3.6 | 0.55 | 0.52 | 0.57 | 845 | 739 | 341633.0 | < 0.001 |
| CB.3.Any | IS.3.SM | 0.64 | 0.62 | 0.67 | 882 | 748 | 424640.0 | < 0.001 |

Vargha and Delaney’s A, 95% confidence intervals, no effect = 0.50

Mann-Whitney-Wilcoxon U test

## Table S.3.7: Stochastic dominance of conspiracy beliefs with regard to reported level of knowledge about COVID-19 from ‘friends and family’

| Conspiracy belief | Source | Est. | Low | High | N1 | N2 | U | p |
| --- | --- | --- | --- | --- | --- | --- | --- | --- |
| CB.3.1 | IS.3.7 | 0.57 | 0.54 | 0.59 | 637 | 971 | 350959.5 | < 0.001 |
| CB.3.2 | IS.3.7 | 0.66 | 0.61 | 0.70 | 173 | 1807 | 204669.0 | < 0.001 |
| CB.3.3 | IS.3.7 | 0.65 | 0.60 | 0.69 | 154 | 1874 | 187489.0 | < 0.001 |
| CB.3.4 | IS.3.7 | 0.59 | 0.56 | 0.63 | 285 | 1586 | 268396.0 | < 0.001 |
| CB.3.5 | IS.3.7 | 0.65 | 0.62 | 0.68 | 268 | 1618 | 282090.0 | < 0.001 |
| CB.3.Any | IS.3.7 | 0.60 | 0.57 | 0.63 | 878 | 749 | 397473.5 | < 0.001 |

Vargha and Delaney’s A, 95% confidence intervals, no effect = 0.50

Mann-Whitney-Wilcoxon U test

## Table S.3.8: Stochastic dominance of health-protective behaviours with regard to reported level of knowledge about COVID-19 from each legacy media source

| Health-protective behaviour | Source | Est. | Low | High | N1 | N2 | U | p |
| --- | --- | --- | --- | --- | --- | --- | --- | --- |
| HPB.3.1 | IS.3.1 | 0.56 | 0.52 | 0.60 | 2003 | 191 | 214456.0 | 0.003 |
| HPB.3.1 | IS.3.2 | 0.53 | 0.48 | 0.57 | 1989 | 190 | 199097.5 | 0.199 |
| HPB.3.1 | IS.3.LM | 0.56 | 0.52 | 0.61 | 2013 | 192 | 217455.0 | 0.003 |
| HPB.3.2 | IS.3.1 | 0.57 | 0.52 | 0.63 | 2078 | 106 | 126518.0 | 0.005 |
| HPB.3.2 | IS.3.2 | 0.55 | 0.50 | 0.60 | 2064 | 105 | 119878.5 | 0.054 |
| HPB.3.2 | IS.3.LM | 0.58 | 0.53 | 0.63 | 2088 | 106 | 128376.0 | 0.004 |
| HPB.3.3 | IS.3.1 | 0.54 | 0.48 | 0.58 | 2063 | 131 | 144472.0 | 0.150 |
| HPB.3.3 | IS.3.2 | 0.43 | 0.38 | 0.48 | 2052 | 126 | 111949.5 | 0.008 |
| HPB.3.3 | IS.3.LM | 0.47 | 0.42 | 0.52 | 2074 | 131 | 127384.0 | 0.220 |
| HPB.3.4 | IS.3.1 | 0.52 | 0.49 | 0.55 | 1867 | 320 | 311276.0 | 0.192 |
| HPB.3.4 | IS.3.2 | 0.48 | 0.45 | 0.52 | 1855 | 317 | 284334.0 | 0.325 |
| HPB.3.4 | IS.3.LM | 0.50 | 0.47 | 0.54 | 1876 | 322 | 303966.5 | 0.851 |
| HPB.3.All | IS.3.1 | 0.54 | 0.51 | 0.56 | 1601 | 561 | 481068.5 | 0.006 |
| HPB.3.All | IS.3.2 | 0.51 | 0.48 | 0.54 | 1594 | 553 | 447648.5 | 0.564 |
| HPB.3.All | IS.3.LM | 0.53 | 0.50 | 0.56 | 1610 | 563 | 478174.0 | 0.046 |

Vargha and Delaney’s A, 95% confidence intervals, no effect = 0.50

Mann-Whitney-Wilcoxon U test

## Table S.3.9: Stochastic dominance of health-protective behaviours with regard to reported level of knowledge about COVID-19 from each social media source

| Health-protective behaviour | Source | Est. | Low | High | N1 | N2 | U | p |
| --- | --- | --- | --- | --- | --- | --- | --- | --- |
| HPB.3.1 | IS.3.3 | 0.44 | 0.40 | 0.48 | 1964 | 188 | 163126.0 | 0.003 |
| HPB.3.1 | IS.3.4 | 0.46 | 0.42 | 0.50 | 1991 | 189 | 173571.5 | 0.059 |
| HPB.3.1 | IS.3.5 | 0.46 | 0.42 | 0.50 | 1962 | 187 | 169577.0 | 0.040 |
| HPB.3.1 | IS.3.6 | 0.47 | 0.43 | 0.50 | 1938 | 186 | 168174.0 | 0.080 |
| HPB.3.1 | IS.3.SM | 0.44 | 0.40 | 0.49 | 2006 | 192 | 170801.0 | 0.008 |
| HPB.3.2 | IS.3.3 | 0.36 | 0.31 | 0.41 | 2037 | 105 | 77366.0 | < 0.001 |
| HPB.3.2 | IS.3.4 | 0.40 | 0.34 | 0.45 | 2065 | 104 | 84862.0 | < 0.001 |
| HPB.3.2 | IS.3.5 | 0.36 | 0.31 | 0.41 | 2035 | 104 | 76251.0 | < 0.001 |
| HPB.3.2 | IS.3.6 | 0.39 | 0.33 | 0.44 | 2013 | 101 | 78379.0 | < 0.001 |
| HPB.3.2 | IS.3.SM | 0.34 | 0.28 | 0.40 | 2081 | 106 | 75160.5 | < 0.001 |
| HPB.3.3 | IS.3.3 | 0.25 | 0.21 | 0.30 | 2025 | 127 | 64990.5 | < 0.001 |
| HPB.3.3 | IS.3.4 | 0.29 | 0.24 | 0.33 | 2050 | 129 | 76471.0 | < 0.001 |
| HPB.3.3 | IS.3.5 | 0.23 | 0.19 | 0.28 | 2024 | 125 | 58259.0 | < 0.001 |
| HPB.3.3 | IS.3.6 | 0.26 | 0.22 | 0.31 | 2002 | 124 | 65744.0 | < 0.001 |
| HPB.3.3 | IS.3.SM | 0.20 | 0.16 | 0.24 | 2067 | 131 | 53855.0 | < 0.001 |
| HPB.3.4 | IS.3.3 | 0.38 | 0.35 | 0.42 | 1829 | 316 | 222449.0 | < 0.001 |
| HPB.3.4 | IS.3.4 | 0.40 | 0.37 | 0.44 | 1854 | 320 | 238209.0 | < 0.001 |
| HPB.3.4 | IS.3.5 | 0.38 | 0.35 | 0.42 | 1832 | 311 | 219485.0 | < 0.001 |
| HPB.3.4 | IS.3.6 | 0.41 | 0.38 | 0.44 | 1806 | 313 | 233592.0 | < 0.001 |
| HPB.3.4 | IS.3.SM | 0.37 | 0.34 | 0.40 | 1869 | 322 | 222309.0 | < 0.001 |
| HPB.3.All | IS.3.3 | 0.39 | 0.37 | 0.42 | 1570 | 550 | 338736.5 | < 0.001 |
| HPB.3.All | IS.3.4 | 0.41 | 0.38 | 0.44 | 1591 | 557 | 363152.5 | < 0.001 |
| HPB.3.All | IS.3.5 | 0.40 | 0.38 | 0.42 | 1573 | 546 | 342731.0 | < 0.001 |
| HPB.3.All | IS.3.6 | 0.42 | 0.40 | 0.45 | 1550 | 543 | 356630.5 | < 0.001 |
| HPB.3.All | IS.3.SM | 0.38 | 0.35 | 0.41 | 1603 | 563 | 342191.5 | < 0.001 |

Vargha and Delaney’s A, 95% confidence intervals, no effect = 0.50

Mann-Whitney-Wilcoxon U test

## Table S.3.10: Stochastic dominance of health-protective behaviours with regard to reported level of knowledge about COVID-19 from ‘friends and family’

| Health-protective behaviour | Source | Est. | Low | High | N1 | N2 | U | p |
| --- | --- | --- | --- | --- | --- | --- | --- | --- |
| HPB.3.1 | IS.3.7 | 0.50 | 0.46 | 0.55 | 2001 | 190 | 191122.0 | 0.896 |
| HPB.3.2 | IS.3.7 | 0.44 | 0.38 | 0.50 | 2076 | 105 | 96429.0 | 0.034 |
| HPB.3.3 | IS.3.7 | 0.33 | 0.29 | 0.39 | 2062 | 130 | 89579.0 | < 0.001 |
| HPB.3.4 | IS.3.7 | 0.40 | 0.37 | 0.43 | 1863 | 321 | 240145.0 | < 0.001 |
| HPB.3.All | IS.3.7 | 0.44 | 0.41 | 0.46 | 1601 | 560 | 393182.5 | < 0.001 |

Vargha and Delaney’s A, 95% confidence intervals, no effect = 0.50

Mann-Whitney-Wilcoxon U test

## Table S.3.11: Odds ratios, conspiracy beliefs vs health-protective behaviours

| Conspiracy belief | Health-protective behaviour | Est. | Low | High | p |
| --- | --- | --- | --- | --- | --- |
| CB.3.1 | HPB.3.1 | 0.73 | 0.52 | 1.03 | 0.060 |
| CB.3.1 | HPB.3.2 | 0.72 | 0.46 | 1.14 | 0.144 |
| CB.3.1 | HPB.3.3 | 0.61 | 0.40 | 0.93 | 0.018 |
| CB.3.1 | HPB.3.4 | 0.59 | 0.44 | 0.77 | < 0.001 |
| CB.3.1 | HPB.3.All | 0.63 | 0.50 | 0.79 | < 0.001 |
| CB.3.2 | HPB.3.1 | 0.41 | 0.26 | 0.65 | < 0.001 |
| CB.3.2 | HPB.3.2 | 0.25 | 0.14 | 0.44 | < 0.001 |
| CB.3.2 | HPB.3.3 | 0.10 | 0.07 | 0.16 | < 0.001 |
| CB.3.2 | HPB.3.4 | 0.27 | 0.19 | 0.39 | < 0.001 |
| CB.3.2 | HPB.3.All | 0.21 | 0.15 | 0.30 | < 0.001 |
| CB.3.3 | HPB.3.1 | 0.28 | 0.18 | 0.44 | < 0.001 |
| CB.3.3 | HPB.3.2 | 0.12 | 0.07 | 0.19 | < 0.001 |
| CB.3.3 | HPB.3.3 | 0.06 | 0.04 | 0.10 | < 0.001 |
| CB.3.3 | HPB.3.4 | 0.20 | 0.14 | 0.30 | < 0.001 |
| CB.3.3 | HPB.3.All | 0.12 | 0.08 | 0.18 | < 0.001 |
| CB.3.4 | HPB.3.1 | 0.39 | 0.27 | 0.57 | < 0.001 |
| CB.3.4 | HPB.3.2 | 0.18 | 0.11 | 0.28 | < 0.001 |
| CB.3.4 | HPB.3.3 | 0.18 | 0.12 | 0.28 | < 0.001 |
| CB.3.4 | HPB.3.4 | 0.33 | 0.24 | 0.45 | < 0.001 |
| CB.3.4 | HPB.3.All | 0.21 | 0.16 | 0.28 | < 0.001 |
| CB.3.5 | HPB.3.1 | 0.37 | 0.25 | 0.55 | < 0.001 |
| CB.3.5 | HPB.3.2 | 0.24 | 0.15 | 0.39 | < 0.001 |
| CB.3.5 | HPB.3.3 | 0.17 | 0.11 | 0.26 | < 0.001 |
| CB.3.5 | HPB.3.4 | 0.35 | 0.26 | 0.48 | < 0.001 |
| CB.3.5 | HPB.3.All | 0.26 | 0.20 | 0.34 | < 0.001 |
| CB.3.Any | HPB.3.1 | 0.45 | 0.31 | 0.64 | < 0.001 |
| CB.3.Any | HPB.3.2 | 0.23 | 0.12 | 0.41 | < 0.001 |
| CB.3.Any | HPB.3.3 | 0.18 | 0.10 | 0.30 | < 0.001 |
| CB.3.Any | HPB.3.4 | 0.44 | 0.32 | 0.58 | < 0.001 |
| CB.3.Any | HPB.3.All | 0.37 | 0.29 | 0.47 | < 0.001 |

Fisher’s exact test, 95% confidence intervals, no effect = 1.00
